# Filtered-orthogonal wavelet division multiplexing (F-OWDM) technique for 5G and beyond communication systems

**DOI:** 10.1038/s41598-022-08248-3

**Published:** 2022-03-17

**Authors:** Ali. F. Almutairi, A. Krishna

**Affiliations:** Electrical Engineering Department, College of Engineering and Petroleum, Kuwait University, P.O. BOX 5969, 13020 Kuwait City, Kuwait

**Keywords:** Electrical and electronic engineering, Information theory and computation

## Abstract

Filtered-orthogonal frequency division multiplexing (F-OFDM) is one of the most protruding multicarrier modulation (MCM) techniques for fifth-generation and beyond wireless communication. However, it possesses a high peak-to-average power ratio (PAPR), which results in its poor performance. Thus, a novel wavelet based MCM technique, namely filtered orthogonal wavelet division multiplexing (F-OWDM), is proposed as an efficient alternative to conventional F-OFDM (C-F-OFDM) to reduce the PAPR. In this model, the traditional Fourier transforms (FT) as used in C-F-OFDM was replaced with Wavelet Transforms. The proposed F-OWDM system does not require a cyclic prefix because of the overlapping sub-carriers in the time and frequency domains. Thus, the F-OWDM system exhibits higher bandwidth efficiency. In this paper, the performance of the F-OWDM system with various wavelets from different wavelet families under an additive white Gaussian noise and flat fading channel is investigated. The PAPR and bit error rate (BER) of the F-OWDM system using Haar, discrete Meyer, bi-orthogonal, symlet, and Daubechies wavelets are analyzed. A comparative study between F-OWDM and C-F-OFDM is also presented. From the results, it is able to prove that F-OWDM has the advantage of lower PAPR and lower bit error rate than the C-F-OFDM system.

## Introduction

Recently, with fifth-generation (5G) and beyond wireless communication systems, there is a perpetual urgency for high-data-rate transmissions due to the rising number of devices and the great demand for multiple services with high reliability and low latency. In comparison to current fourth-generation (4G) long-term evolution/advanced (LTE/LTE-A) systems, 5G technologies are expected to handle innovative applications such as enhanced mobile broadband (eMBB), massive machine-type communications (mMTC), and ultra-reliable, low-latency communications (URLLC)^1,2^. Moreover, they are expected to enhance the scope of current communication networks, in parallel with unique progress, in this field.

The existing 4G systems considered the cyclic prefix-orthogonal frequency division multiplexing (CP-OFDM) technique as the factual waveform owing to its effectiveness to fray fading channel characteristics and low complexity. However, CP-OFDM uses rectangular pulses, which makes it more susceptible to frequency and time offset and develop high out-of-band (OoB) leakage. This degrades the spectrum efficiency when many pieces of user equipment are operating at one location. Moreover, if the transmission condition becomes imperfect, it caused severe performance degradation because inter-carrier interference (ICI) spreads over a wide sub-carrier range. Due to these reasonable shortcomings, orthogonal frequency division multiplexing (OFDM) is not represented as an adequate technique for 5G communications and hence, researchers persuade to find appropriate waveforms for 5G and beyond systems^3,4^. To eliminate these disadvantages, new alternative waveforms, such as universal filtered multi-carrier (UFMC), filtered-OFDM (F-OFDM), generalized frequency division multiplexing (GFDM) and filter bank multi-carrier (FBMC) are being proposed as potential candidates for 5G and beyond communications^5–11^.

F-OFDM is one of the most promising waveforms designed for 5G and beyond communication systems^6,12,13^. The F-OFDM is a sub-band filtering multi-carrier waveform that uses a time-domain filtering technique after CP-OFDM modulation^6,14–16^. CP-OFDM based on fast Fourier transform (FFT) requires insertion of a cyclic prefix (CP) to have the delay spread of the channel longer than the channel impulse response to eliminate inter-symbol-interference (ISI) and has high tolerance against multipath propagation and fading. However, due to the use of the CP, the spectral efficiency of CP-OFDM deteriorate. The conventional F-OFDM (C-F-OFDM) completely possesses the CP-OFDM process. Therefore, the spectral efficiency of F-OFDM is similar to that of CP-OFDM. Thus, to improve the efficiency of C-F-OFDM system, wavelet-transform (WT) based OFDM is used before sub-band filtering instead of the conventional CP-OFDM (C-OFDM).

The WT has been introduced as one of the most extrusive signal analysis tools that are generally used for speech and image compression analysis. However, in the wireless communication field, discrete WT (DWT) has been proposed as an alternative approach to the multicarrier modulation (MCM) technique^17–20^. An OFDM technique based on wavelet filter banks, called orthogonal wavelet division multiplexing (OWDM) has been utilized in digital communication to improve the system robustness to noise and adjacent channel interference^21–27^. It was proved that OWDM is one of the best MCM techniques that can provide much higher spectrum efficiency, reduce the ISI and ICI, and provide the best PAPR and BER performance in comparison with C-OFDM (Please note: In this article, we use conventional OFDM (C-OFDM) for DFT-OFDM (discrete Fourier transform-OFDM) and conventional F-OFDM (C-F-OFDM) for F-DFT-OFDM.)^28–30^. This motivates us to investigate this method on the C-F-OFDM system for improving the system performance.

The conform of OWDM based MCM technique for the future 5G applications is scarcely researched. Thus, in this paper, we tried to analyze the benefits of OWDM based system for 5G and beyond applications. The contributions of the paper can be summarized as follows:

- In this paper, we have proposed a novel wavelet based MCM technique called Filtered-orthogonal wavelet division multiplexing (F-OWDM), instead of C-F-OFDM, by replacing Fourier transform (FT) with WT. This is an extension of existing orthogonal wavelet division multiplexin (OWDM) system by additional per-sub-band filtering operation. Hence, the proposed F-OWDM can be used as an effective method for 5G communication which is related to the inverse discrete wavelet transform (IDWT) rather than inverse Fast Fourier transform (IFFT). Different wavelet filters from various wavelet families are used for the analysis to identify the suitable wavelet for our MCM technique.
- Then, we have analyzed the PAPR performance of proposed F-OWDM system and compared the results with C-OFDM and C-F-OFDM for different modulation indexes. In addition, we investigated the BER performance of the proposed model under flat fading channel having Rayleigh distribution. We have compared the BER performance of orthogonal and Biorthogonal wavelet based F-OWDM system. we made a comprehensive comparison between the F-OWDM and C-OFDM system. Finally, the spectral efficiency of proposed model is examined and compared with C-OFDM and C-F-OFDM.

As mentioned earlier, the authenticity of the C-OFDM is confined due to the time-varying behavior of the channel. This may introduce ICI and escalate deceptions in channel modeling. Thus, in our prospective model, we use DWT, which provides time-and frequency-domain analysis of the signal (i.e. multiresolution analysis). Moreover, due to the overlapping property of DWT, there is no need of a CP in our proposed F-OWDM system. The removal of the CP enables the WT to make full use of its spectral efficiency. This leads to improving the system performance in F-OWDM compared to C-F-OFDM. Thus, the proposed 5G waveform able to deal with the major limitations of C-F-OFDM such as the reduction in transmission throughput due to the use of CP, high PAPR, and OoB emission.

## Wavelet analysis

WT based analysis (wavelet analysis) is one of the recent advancements in the field of engineering and communication even if it was proposed by Alfred Haar in 1909. The Wavelet analysis is considered as a possible alternative to Fourier analysis because of its ability to analyze rapidly changing transient signals^31–33^. Wavelets are effective for investigating aperiodic, noisy signals in both time and frequency domains simultaneously. These are small waveforms which are time limited or exist only for a short period of time^34^. The wavelet analysis constitute two general operations such as decomposition (DWT) and reconstruction (IDWT). The short duration wavelet is superimposed to the signal under consideration for a short duration of time and decompose them to useful form. This process is called wavelet transform (WT). The method of transforming the decomposed signal to original wave is called inverse wavelet transform (IWT)^34^.

Each wavelet of the discrete wavelet family consists of two basis functions called the mother wavelet ($\psi (t)$) and the scaling function ($\phi (t)$) as shown in (1) and (2), respectively. Thus, the process of discrete scaling and translation of $\psi (t)$ can be represented as in (3):

1 $$\begin{aligned} \psi (t)= & {} 2^{\textit{j}/2}\psi (2^{\textit{j}}t) \end{aligned}$$

2 $$\begin{aligned} \phi (t)= & {} 2^{\textit{j}/2}\phi (2^{\textit{j}}t) \end{aligned}$$

3 $$\begin{aligned} \psi _{\textit{j,k}}(t)= & {} \frac{1}{\sqrt{S_{0}^{\textit{j}}}} \psi \left( \frac{t-\textit{k}\tau _{0}S_{0}^{\textit{j}}}{S_{0}^{\textit{j}}}\right) \end{aligned}$$

where *j* and *k* are integers which indicate the scale and translation indices, $S_{0}>1$ is a fixed dilation step and $\tau _{0}$ is the translation factor^30,35^. The important features of a signal can be better described using the scaling functions and wavelets which are orthogonal to each other^32^. This means, that

4 $$\begin{aligned} \left\langle \phi _{\textit{j,k}}(t) , \psi _{\textit{j,l}}(t)\right\rangle =\int \phi _{\textit{j,k}}(t) \psi _{\textit{j,l}}(t) \textit{dt }=0 \end{aligned}$$

for all $$j,k,l \in {\mathbb {Z}}$$.

Moreover, the scaling function and wavelet functions can be expressed by the weighted sum of the time-shifted scaling functions as shown in (5) and (6).

5 $$\begin{aligned} \phi (t)= & {} \sum _{n} g\,(n) \sqrt{2}\phi (2t-n),n\in Z \end{aligned}$$

6 $$\begin{aligned} \psi (t)= & {} \sum _{n} h\,(n) \sqrt{2}\phi (2t-n),n\in Z \end{aligned}$$

where the term *g*(*n*) represents the scaling filter coefficient and *h*(*n*) represents the wavelet filter coefficient.

After scaling and translation of the time variable in (5), it can be expressed as in (7),

7 $$\begin{aligned} \begin{aligned} \phi (2^{j}t-k)=&\sum g\,(n) \sqrt{2}\phi (2(2^{j}t-k)-n) \\ =&\sum g\,(n) \sqrt{2}\phi (2^{j+1}t-2k-n) \end{aligned} \end{aligned}$$

Let $m=2k+n$, and then (7) can be represented as in (8),

8 $$\begin{aligned} \begin{aligned} \phi (2^{j}t-k)=&\sum _{m} g\,(m-2k) \sqrt{2}\phi (2^{j+1}t-m) \end{aligned} \end{aligned}$$

Furthermore, the relation between the wavelet and scaling filter coefficients can be represented as follows^32^:

9 $$\begin{aligned} h\,(n)=(-1)^{n}\,g\,(1\,-\,n) \end{aligned}$$

If the scaling filter coefficient has a finite even number of length $\mathbf{N }$, then (9) can be modified as shown in (10)

10 $$\begin{aligned} h\,(n)=(-1)^{n}\,g\,(N\,-\,1\,-\,n) \end{aligned}$$

Then, by using a combination of these scaling and wavelet functions, a large class of signals, i.e., $f(t)\in L^{2}({\mathbb {R}})$, can be represented as in (11)^32^:

11 $$\begin{aligned} f(t)= \sum _{k} c_{j_{0}}(k) \phi _{j_{0},k}(t) + \sum _{k} \sum ^{\infty }_{j=j_{0}} d_{j}(k)\psi _{j,k}(t) \end{aligned}$$

Equation (11) can be modified as shown in (12)

12 $$\begin{aligned} f(t)= \sum _{k} c_{j}(k) 2^{\textit{j}/2}\phi (2^{\textit{j}}t-k) + \sum _{k} d_{j}(k)2^{\textit{j}/2}\psi (2^{\textit{j}}t-k) \end{aligned}$$

In (12), the first summation gives the low-resolution approximation (approximate coefficient) of the function, and the second summation gives the detailed approximation (detailed coefficient) of the signal. The coefficients in (12) are termed DWT of *f*(*t*) and can be evaluated by inner products as shown in (13).

13 $$\begin{aligned} c_{j}(k) =\left\langle f(t) , \phi _{\textit{j,k}}(t)\right\rangle =&\int f(t) \phi _{\textit{j,k}}(t) \textit{dt} \\ =&\int f(t)~ 2^{\textit{j}/2}\phi (2^{\textit{j}}t-k) \textit{dt} \end{aligned}$$

By using (8), and altering the order of the sum and integral, (13) can be rewritten as follows:

14 $$\begin{aligned} c_{j}(k) =&\int f(t)~ 2^{\textit{j}/2} \sum _{m} g\,(m-2k) \sqrt{2}\phi (2^{j+1}t-m) \textit{dt} \end{aligned}$$

15 $$\begin{aligned} c_{j}(k) =&\sum _{m} g\,(m-2k) \int g(t) 2^{(j+1)/2} \phi (2^{j+1}t-m) \textit{dt} \\ =&\sum _{m} g\,(m-2k) c_{j+1}(m) \end{aligned}$$

Similarly, for the wavelet coefficient

16 $$\begin{aligned} d_{j}(k) =\left\langle f(t) , \psi _{\textit{j,k}}(t)\right\rangle =&\int f(t) \psi _{\textit{j,k}}(t) \textit{dt }\\ =&\int f(t)~ 2^{\textit{j}/2}\psi (2^{\textit{j}}t-k) \textit{dt} \end{aligned}$$

Thus

17 $$\begin{aligned} \begin{aligned} d_{j}(k) =\sum _{m} h\,(m-2k) c_{j+1}(m) \end{aligned} \end{aligned}$$

**Figure 1:**
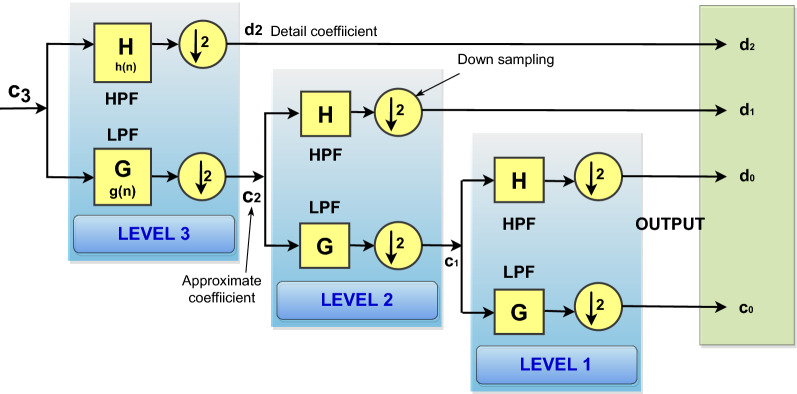
Representation of three-level discrete wavelet transform.

From (15) and (17), it is clear that the DWT coefficients are computed by taking the weighted sum of the DWT coefficients of higher scale $(j\,+\,1)$. To obtain the wavelet coefficient ($d_{j}(k)$) at scale *j*, the wavelet function coefficient *h*(*n*) is convolved with the scaling function at scale $j+1$, followed by down-sampling by a factor of **2**. Similarly, for the scaling coefficient ($c_{j}(k)$), the scaling function coefficient *g*(*n*) is convolved with the scaling function at scale $j+1$, followed by down-sampling by a factor of **2**. The general representation of decomposition coefficients are explained in (15) and (17) and its implementation is demonstrated in Fig. 1.

### Decomposition

Figure 1 represents the three stage two-channel filter bank DWT decomposition analysis. The decomposition procedure is accomplished through a series of filtering and downsampling processes as shown in the figure. For an input signal of length $\mathbf{N }$, the WT consists of $\log _{2} (N)$ decomposition levels (stages). Each stage has a combination of filter and sub-sampling operations and is known as decimation. At each stage, the input signal is decomposed into two coefficients: approximate coefficients ($c_{j}\,(k)$) and detailed coefficients ($d_{j}\,(k)$).

It is evident from Fig. 1 that, at the first level, the original signal is decomposed through a half-band low-pass filter (LPF) (scaling filter, $\mathbf{G }$) and a half-band high-pass filter (HPF) (wavelet filter, $\mathbf{H }$). The $\mathbf{G }$ filter excludes the frequencies above a particular level, and then the filered signal is down-sampled by two, to generate $c_{j}\,(k)$ as in (14). Similarly, the $\mathbf{H }$ filter removes all signals below the half-band frequencies with the same scale. Then, the filtered signal is sub-sampled by two to generate $d_{j}\,(k)$ as illustrated in (16). Then, at the second stage, the lower frequency band is again divided into low pass and high pass band signals and the procedure will be repeated. At the final stage, the output will be a combination of the detailed coefficients of previous stages and the approximate coefficient of the final stages, as shown in Fig. 1.

### Reconstruction

The reconstruction of the original signal from the scaling and wavelet coefficients is know as IDWT or reconstruction operation. The reconstruction of the original signal is obtained through a series of operations such as upsampling, and series filtering. The IDWT operation can be illustrated as in (12). Then, substitute (7) into (12) and we get the following:

18 $$\begin{aligned} \begin{aligned} f(t)&= \sum _{k} c_{j}(k) 2^{\textit{j}/2}\left[ \sum _{n} g\,(n) \sqrt{2}\phi (2^{j+1}t-2k-n) \right] \\&\quad +\, \sum _{k} d_{j}(k)2^{\textit{j}/2} \left[ \sum _{n} h\,(n) \sqrt{2}\psi (2^{j+1}t-2k-n) \right] \end{aligned} \end{aligned}$$

Then, multiplying (18) by $\phi (2^{j+1}t-k)$ and taking the integral will give the reconstruction coefficient as in (19)

19 $$\begin{aligned} \begin{aligned} c_{j+1}(k)= \sum _{m} c_{j}(m) g\,(k-2m) + \sum _{m} d_{j}(m) h\,(k-2m) \end{aligned} \end{aligned}$$

**Figure 2:**
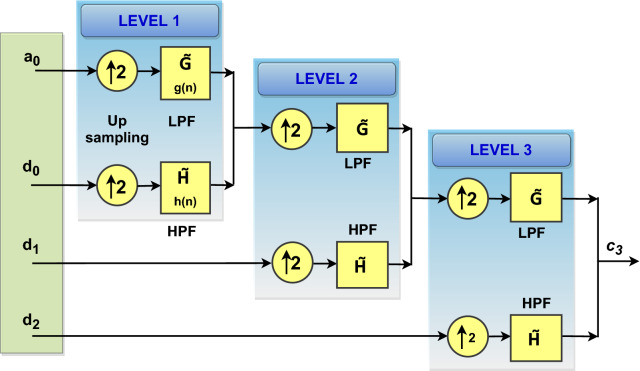
Three-level inverse discrete wavelet transform.

Figure 2 shows the three-stage(level-3) synthesis filter bank operation or IDWT procedure. The reconstruction procedure consists of a combination of inverse scaling and wavelet filter operations (${\tilde{\mathbf{G}}}$ and ${\tilde{\mathbf{H}}}$) along with up-sampling operation as illustrated in Fig. 2^32^.

During the reconstruction procedure, there is a progression of up-sampling and filtering operations being carried out. The scaling coefficients $c_{j}\,(k)$ and wavelet coefficients $d_{j}\,(k)$ at scale *j* are first up-sampled by a factor of $\mathbf{2 }$. Then, $c_{j}\,(k)$ and $d _{j}\,(k)$ are convolved with LPF (G̃ ) and HPF (H̃ ) respectively. Finally, the two filtered signals are added to form the ($j+1$) level scaling DWT coefficients ($c_{j+1}$). However, this procedure can be repeated to different levels similar to the decomposition level to reconstruct the original signal.

## C-F-OFDM system model

C-F-OFDM is one of the most promising 5G waveform candidates, that can fulfill various 5G performance requirements such as efficient spectrum utilization, simple channel equalization, low latency and reliability^12^. In C-F-OFDM system, the entire bandwidth is divided into several sub-bands that are similar to those in the C-OFDM system. The output of each C-OFDM signal then passed through spectral shaping filters to generate C-F-OFDM signals. It is worth to mention that, the subcarriers in C-F-OFDM systems are orthogonal and overlapped with each other^16^.

**Figure 3:**
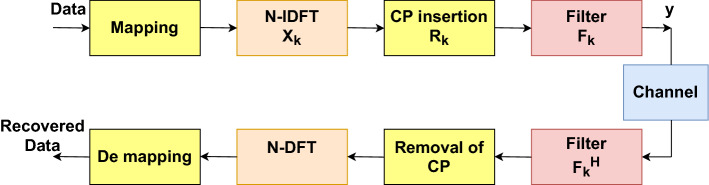
Block diagram of the C-F-OFDM system.

In the C-F-OFDM system, the message symbols are modulated using quadrature amplitude modulation (QAM) and the modulated data symbols of individual sub-bands are passed to the N-point IDFT blocks to obtain the time-domain modulated signal that has a length of *N* samples as illustrated in (20). Later, the CP of length $L_{CP}$ will be added to the time-domain signal followed by filter $f_{i}$ of length $L_{F}$. The block diagram of the C-F-OFDM system for k^th^ sub-band is shown in Fig. 3.

20 $$\begin{aligned} x(n)=IDFT\left[ \mathbf {X_{k}}\right] =\frac{1}{\sqrt{N}}\sum ^{N-1}_{k=0}X_{k}e^{j2\pi nk/N} \end{aligned}$$

Finally, the transmitted signal vector can be represented as (21).

21 $$\begin{aligned} \mathbf {y_{tr,k}}= \mathbf {R_{k} d_{k}X_{k}F_{k}} \end{aligned}$$

where $R_{k}$, $d_{k}$, $F_{k}$, and $X_{k}$ represent the CP insertion matrix, the data vector, sub-band filtering matrix and N-point IDFT matrix, respectively. Later, at the receiver sub-band filtering of the received signal is performed using a matched filter, and then, the entire operations performed at the transmitter are repeated backward to regenerate the original information (Note that, the system model for conventional F-OFDM will not discussed in detail as it is available in^16^.).

## Proposed F-OWDM system model

F-OWDM is an MCM system based on the wavelet basis, where the carriers are represented using the wavelet functions ($\psi _{j,k}$), where $j \in \llbracket J_{0}, J-1\rrbracket$, $k \in \llbracket 0,2^{j-1} \rrbracket$, and the scaling functions ($\phi _{J_{0},k}$). In the wavelet-based MCM system, each sub-carrier will have its own time and frequency resolutions and the system will depend on filter bank instead of Fourier transform to generate the orthogonal sub-carriers^18^.

**Figure 4:**
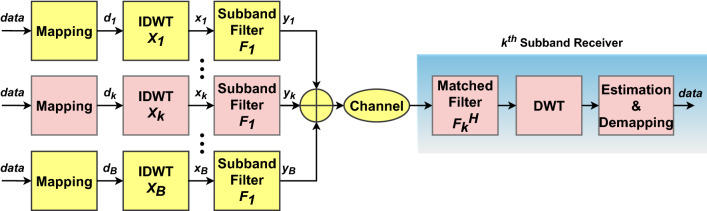
Block diagram of the proposed F-OWDM system for the k^th^ sub-band.

The block diagram of the proposed F-OWDM system is shown in Fig. 4. As illustarted in the figure, in the F-OWDM system, the entire bandwidth is divided into several sub-bands ($\mathbf{B }$), and each sub-band consists of a number of sub-carriers ($M_{B}$) such that $M=BM_{B}$, where *M* is the total number of sub-carriers as shown in Fig. 4. However, For the sake of simplicity, we have considered only single sub-band (k^th^ sub-band) in this research.

At the transmitter side, the data can be represented in vector form as $\mathbf{b }$ = [$b_{1}$; $b_{2}$; $\cdots$; $b_{B}$] $\in$ ${\mathbb {C}}$ $^{M \times 1}$, where ${b_{k}}$ is the signal transmitted in the kth sub-band as represented in (22).

22 $$\begin{aligned} b_{k}=[b_{k}(1),~ b_{k}(2), \cdots , b_{k}(M_{B})]^T \in {\mathbb {C}} ^{M_{B} \times 1} \end{aligned}$$

In each sub-band, the data bits are mapped using an QAM technique and processed with IDWT instead of IFFT. As mentioned in the previous section, the IDWT block apparently abide of different levels of up-samplers, followed by low pass and high pass filter banks. Thus, at the IDWT block, the QAM modulated signal is up-sampled with a factor of ${\mathbf {2}}$, and then convoluted with the synthesis LPF coefficient. This LPF coefficient is known as the scaling coefficient or approximate coefficients. Meanwhile, for the detailed coefficient, zero padding is applied and the zero padded signal undergoes circular convolution with the synthesis HPF coefficient or wavelet filter coefficient. This procedure can be repeated to different levels to synthesis the original signal as shown in Fig. 2. A detailed explanation of the IDWT procedure is available in the previous “Reconstruction” section. The applicability of the filter bank makes the WT anyhow barely complex than the FT in the C-F-OFDM.

Later, the outputs of the HPF and LPF branches are subsequently added and then passed through a filter $f_{i}$ of length *L*. The potential benefit of applying WT is that the overlapping behavior of wavelet properties conserves the orthogonality of the output IDWT signal at the transmitter. Hence, there is no requirement of CP in F-OWDM to fix the orthogonality loss due to time dispersion effect. The ideal LPF that can be used in a F-OWDM system to have a flat passband over the sub-carriers in the sub-band is a filter with a rectangular frequency response, which ensures smooth transitions for both ends^8,12,36^. The rooted raised cosine (RRC) window function meets the flexibility requirement of the F-OWDM better than other window functions^12^. Finally, these time-domain output signals form the filters of different sub-bands add together to generate the F-OWDM base-band signal, as shown in Fig. 4. The F-OWDM base band signal is then propagated through wireless channel. In this research, an additive white Gaussian noise (AWGN) channel, and Rayleigh fading channel are used for the analysis.

At the receiver side, the corresponding modulation operations are appropriately reversed to decode the original information. The received signal will be distorted due to the effect of channel and hence Zero-Forcing (ZF) equalizer is used to restore the signal after the channel. Formerly, to retrieve the desired information, filtering of the received signal from the channel is performed using a matched filter. The resulting signal is then applied to the DWT operation. The DWT block used at the receiver consists of low pass and high pass filter banks and is used to recover the reconstructed signals. Hence, the DWT block at the receiver is known as “decomposition filters bank”. The decomposition procedure at the receiver is the inverse process of wavelet reconstruction operation at the transmitter. The output of the matched filter is decomposed into HPF and LPF corresponding to the detailed and approximate coefficients and down sampled by ${\mathbf {2}}$. The down sampling operation helps to diminish the number of coefficients required to reconstruct the signal.

The output of the LPF or the approximate coefficient is further processed using M-QAM demodulation to decode the original information. However, the HPF coefficient or the detailed coefficient does not contain any information and hence it will be discarded. It is worth to mention that, the level of the decomposition operation must be the same as that of the reconstruction filter level. Moreover, the same wavelet family that was used at the transmitter must be used at the receiver for better reconstruction of the original information.

### Wavelet family

As we know, number of wavelet families are available in literature and can classify them into two categories: (a) orthogonal and (b) biorthogonal. The coefficients of orthogonal filters are real numbers and the filters are of the same length and are not symmetric. However, for the biorthogonal wavelet, the LPF and the HPF do not have the same length. The LPF is always symmetric, while the high pass filter could be either symmetric or anti-symmetric. The coefficients of the filters are either real numbers or integers^37–39^. Thus, the choice of wavelet functions depends on the applications. In this paper, we use 5 different wavelet families for the analysis. A brief explanation of the used wavelet families is available in the following section.

**Figure 5:**
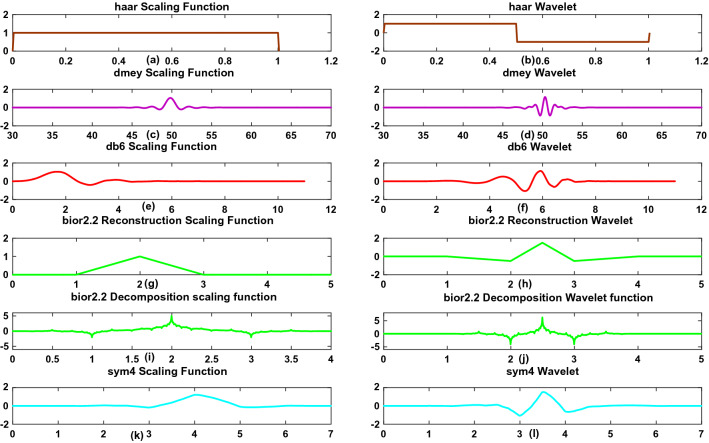
Scaling and wavelet functions of various wavelet families.

#### Haar wavelet

The Haar ( presented “‘*haar*”’ hereafter) wavelet function, which has been discovered by a Hungarian scientist, is the oldest and simplest wavelet function. The *haar* wavelet is a memory efficient wavelet and preserves the energy of the signal. The *haar* wavelet is a member of the Daubechies family and is the first-order Daubechies wavelet (db1). The mother *haar* wavelet and the *haar* scaling function are presented in Fig. 5a and b, and can be expressed as follows:

$$\begin{aligned} \psi (t)= & {} {\left\{ \begin{array}{ll} 1,&{} \text {for}~ 0\le t< 1/2,\\ -1,&{} \text {for}~ 1/2 \le t< 1,\\ 0,&{} \text {otherwise} \end{array}\right. }\\ \phi (t)= & {} {\left\{ \begin{array}{ll} 1,&{} \text {for}~ 0\le t < 1,\\ 0, &{} \text {otherwise} \end{array}\right. } \end{aligned}$$

From Fig. 5, it is clear that, the *haar* wavelet is discontinuous and resembles a step function.

#### Dmey wavelet

The Meyer wavelet is a frequency-band-limited orthogonal wavelet, which has been proposed by Yves Meyer in 1985. The discrete Meyer (presented “‘*dmey*”’ hereafter) wavelet is the finite impulse response (FIR) filter based approximation of the Meyer wavelet. Figure 5c and d, show the *dmey* scaling function and wavelet function.

#### Daubechies wavelet

The Daubechies wavelets are orthogonal and biorthogonal wavelets and represented by *dbN*, where, *N* is the order of the family. The Daubechies wavelets are not symmetrical. Figure 5e and f show the *db*6 scaling function and wavelet function.

#### Biorthogonal wavelet

Biorthogonal wavelets are a family of wavelets that display linear phase and biorthogonal characteristics. By using biorthogonal wavelets ,exact reconstruction of the signals is possible with FIR filters. Biorthogonal wavelets are the generalization of orthogonal wavelet systems which are more flexible and easy to design. They use two wavelets and two scaling functions, one for decomposition and the other for reconstruction operation, and hence, different multiresolution analysis is possible. It is represented by *biorNr*.*Nd*, where, *Nr* and *Nd* represent the order of the family, where *r* is for reconstruction and *d* is for decomposition. In this paper, we use the *bior*2.2 wavelet, and its corresponding decomposition and reconstruction wavelet and scaling function are shown in Fig. 5g and h and i and j, respectively.

#### Symlet wavelet

Symlet wavelets, represented as *symN*, are known as Daubechies least-asymmetric wavelets with the highest number of vanishing moments. Daubechies made modification to *dbN* wavelets to make them symmetric and simple. Thus, *symN* are more symmetric than the *dbN* wavelets. However, *symN* have all the other properties similar to those of *dbN*, such as orthogonalilty and biorthogonality. Figure 5k and l, shows the *sym*4 scaling and wavelet functions.

## Results and analysis

To conduct an impressive performance evaluation of the F-OWDM system, the input data is modulated using the M-QAM technique. For the effective performance analysis of the proposed system, we have studied different parameters such as, power spectral density (PSD), the complementary cummulative distribution function (CCDF) of the PAPR, and BER vs. $E_{b}/N_{0}$ performance. We utilize the 3rd Generation Partnership Project (3GPP) LTE-10 MHz radio frame structure with the number of sub carriers considered in the simulations being $N=1024$. The sub-carrier spacing is 15 kHz, and there are, 600 occupied sub-carriers, which are divided into 50 sub-bands of 12 sub-carriers each. Table 1 summarizes the F-OWDM parameters derived from the LTE system parameters. In this study, we have analyzed performance of F-OWDM system over an AWGN and a multipath Rayleigh fading channel with 10 channel taps and compared it with the performance of C-F-OFDM system to evaluate the advantages of F-OWDM over them. The results of Monte-Carlo simulations being averaged over 10000 F-OWDM blocks.

**Table 1:** Simulation parameters of the F-OWDM system.

Size of FFT (N)	1024
Total number of sub-carriers (*M*)	600
Modulation method (*q*)	M-QAM (4 QAM, 16 QAM, 64 QAM)
Number of sub-bands (*B*)	50
The number of sub-carriers in each sub-band ($M_{B}$)	12
Filter	Root Raised Cosine
Filter length	513
Channel	AWGN and Rayleigh fading
wavelets	$``haar''$, $``dmey''$, $``db6''$, $``bior2.2''$, $``sym4''$

### Performance analysis of the F-OWDM system

This section represents the analysis of the different parameters for the analysis of the performance of the F-OWDM system and compares it with the C-F-OFDM system and C-OFDM technique.

#### PSD

**Figure 6:**
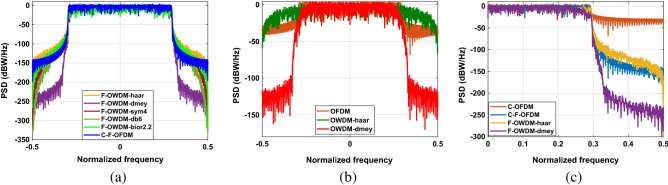
(**a**) Comparison of PSDs of the proposed F-OWDM technique using different wavelets. (**b**) comparison of PSDs of *dmey*-OWDM and *haar*-OWDM with C-OFDM. (**c**) comparison of PSDs of *dmey*-F-OWDM and *haar*-F-OWDM with C-F-OFDM.

PSD is used as a significant precedent in the interpretation of the spectral efficiency of a particular waveform technique. Moreover, PSD symbolizes the bandwidth efficiency of the proposed F-OWDM technique and the adjacent channel interference due to side lobe effects. In this section, we compare the PSD performance of the F-OWDM technique with those of the C-F-OFDM, C-OFDM, and OWDM techniques. The PSD is approximated along a periodogram method with a rectangular window using MATLAB. The PSDs of F-OWDM for different wavelets are displayed in Fig. 6a, and compared with those of C-F-OFDM. From Fig. 6a, it is clear that, all the F-OWDM systems have better PSD characteristics. However, *dmey*-F-OWDM has attractive PSD characteristics compared to all other members of the wavelet family and C-F-OFDM system.

Figure 6b illustrates the comparison of the PSDs of the OWDM system with the C-OFDM system. From the figure, it is clear that the width of the main lobe of the PSD of *haar*-OWDM is almost 50% larger than that the C-OFDM system. It is also worth to mention that, the PSD of the *haar* wavelet OWDM techniques suffers from very large side lobes and shows extremely degraded PSD characteristics. Because of these poor spectral characteristics, *haar*- OWDM is not considered as the best choice for many applications. Contrary to this, *dmey*-OWDM shows better PSD characteristics compared to C-OFDM and is comparable to C-F-OFDM. Figure 6c shows the effect of filtering on the PSD of the *haar* and *dmey* OWDM system. It is evident from the analysis that, *dmey*-F-OWDM has better PSD compared to its counterparts and C-F-OFDM. Thus, the selection of a relevant wavelet family is constrained by the trade-off between PAPR characteristics and PSD performance. Hence, in the next section, we will analyze the PAPR characteristics of the F-OWDM system.

#### PAPR

An extensive shortcoming of MCM techniques is their high Peak-to-Average Power Ratio. The PAPR of the F-OWDM signal is defined as the ratio of the peak power of the F-OWDM signal to its corresponding mean power, which can be expressed as shown in (23):

23 $$\begin{aligned} PAPR=\frac{max(|y(n)|^2)}{E[|y(n)|^2]}, \end{aligned}$$

where $y(n)$ represents the F-OWDM transmitted signal.

**Figure 7:**
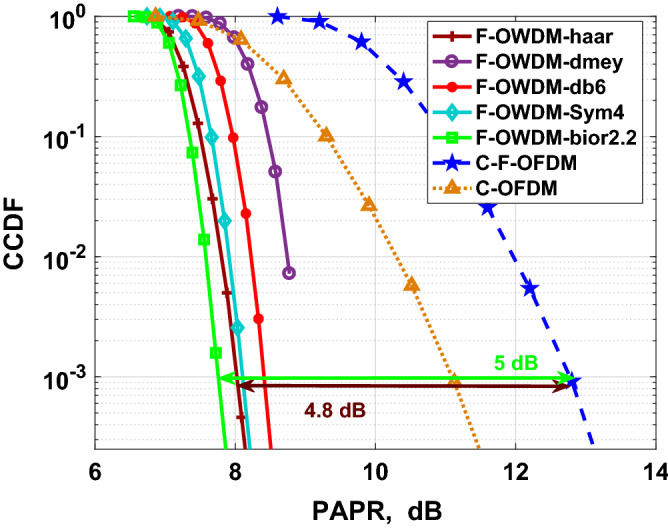
Comparison of PAPRs of F-OWDM with C-F-OFDM and C-OFDM using 4-QAM modulation.

**Figure 8:**
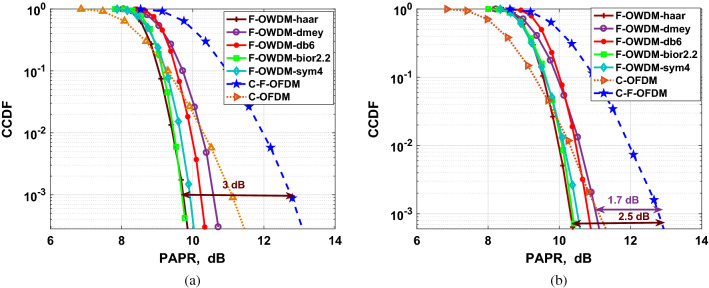
Comparison of PAPRs of F-OWDM with C-F-OFDM and C-OFDM (**a**) using 16-QAM. (**b**) using 64-QAM.

In this section, we analyze the PAPR of the F-OWDM systems based on various parameters, such as modulation schemes, the number of sub-carriers, and decomposition level (*NL*) and then compare the PAPR characteristics of F-OWDM with those of C-F-OFDM systems. To evaluate the PAPR performance, the CCDF of the PAPR is simulated, which shows the probability that the PAPR exceeds a particular threshold value. Figures 7 and 8 show the effect of different modulation constellation mapping on the PAPR of the F-OWDM system.

Figure 7 shows the CCDF characteristics of the proposed F-OWDM system for 4-QAM modulation. The figure shows the comparison of the proposed technique with the C-F-OFDM and C-OFDM systems. From Fig. 7, it is evident that the PAPR of the F-OWDM system is better than those of the C-F-OFDM and C-OFDM systems. Different wavelets are used for the analysis as given in Table 1. It is clear from the analysis that, the *bior*2.2 and *haar* wavelets provide better PAPR performance compared to C-F-OFDM. The *bior*2.2-F-OWDM provides a PAPR improvement of 5 dB compared to the C-F-OFDM system. Moreover, the PAPR of *haar*-F-OWDM decreases to 8 dB at $10^{-3}$ CCDF and hence improves to 4.8 dB at $10^{-3}$ CCDF compared to that of the C-F-OFDM system.

The CCDF plot of the proposed F-OWDM technique for 16-QAM modulation is shown in Fig. 8a. As depicted in the figure, for 16-QAM modulation, the PAPR of *bior*2.2-F-OWDM and *haar*-F-OWDM shows better performance and is less by 3 dB at $10^{-3}$ CCDF compared to that of C-F-OFDM. Figure 8b shows the PAPR comparison of the F-OWDM system with the C-F-OFDM and C-OFDM system for 64-QAM modulation. From Figs. 7, and 8, it is clear that the PAPR of the F-OWDM system surpasses the PAPR characteristics of the C-F-OFDM and C-OFDM systems without any PAPR reduction technique.

**Figure 9:**
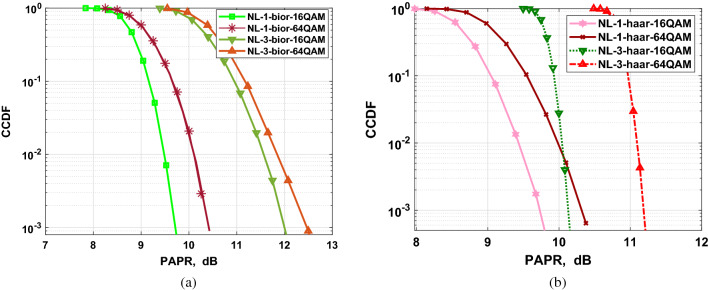
Comparison of PAPRs of F-OWDM using multilevel wavelet transform (**a**) *bior*2.2-F-OWDM (**b**) *haar*-F-OWDM.

Thus, from the PAPR performance analysis of the F-OWDM system for different modulation indexes (4-QAM, 16-QAM, and 64-QAM), it is clear that the PAPR of *haar*-F-OWDM and *bior*2.2-F-OWDM system is less by 4.8 (5), 3 (3) and 2.5 (2.6) dB compared with those of C-F-OFDM, respectively. It is also evident from our analysis that, unlike C-F-OFDM and C-OFDM, the PAPR characteristics of the F-OWDM system depend on the modulation index used.

Finally, to study the effect of multilevel decomposition on PAPR analysis, a 3-level (NL=3) reconstruction and decomposition block is used. Figure 9 shows the comparison of the PAPR characteristics of the *bior*2.2-F-OWDM and *haar*-F-OWDM system for different decomposition levels (NL=1 and NL=3). From the figure, it is clear that the PAPR performance of the F-OWDM system depends on the number of decomposition levels of the WT. When the level of decomposition increases from $NL= 1$ to $NL=3$, the PAPR characteristics degraded, as illustrated in Fig. 9. This is due to the fact that the MCM system is considered as a single carrier system in every decomposition level. This means that, the PAPR performance for a single level ($NL=1$) F-OWDM system is exceptional compared to that for a multilevel system since single level system contains a smaller number of signal analysis than the multilevel system.

#### Bit error rate

In this section, the simulation result of the BER characteristics of the F-OWDM system is estimated against the C-F-OFDM system to analyze the performance enhancement of the proposed system. Here, we compare the BER performances at different SNR values for the F-OWDM and C-F-OFDM systems for the 4-QAM, and 16-QAM modulation techniques. Comparison between the BER performance of *haar* , *dmey*, *bior*2.2, *db*6, and *sym*4 wavelet families based F-OWDM system, over both AWGN and fading channel is provided and compared the result with C-F-OFDM system.

#### AWGN

**Figure 10:**
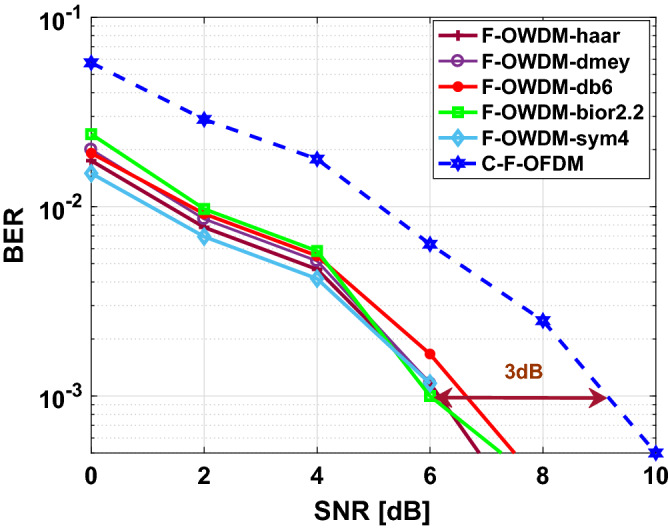
Comparison of BER of the F-OWDM and C-F-OFDM signals based on the 4-QAM modulation technique.

**Figure 11:**
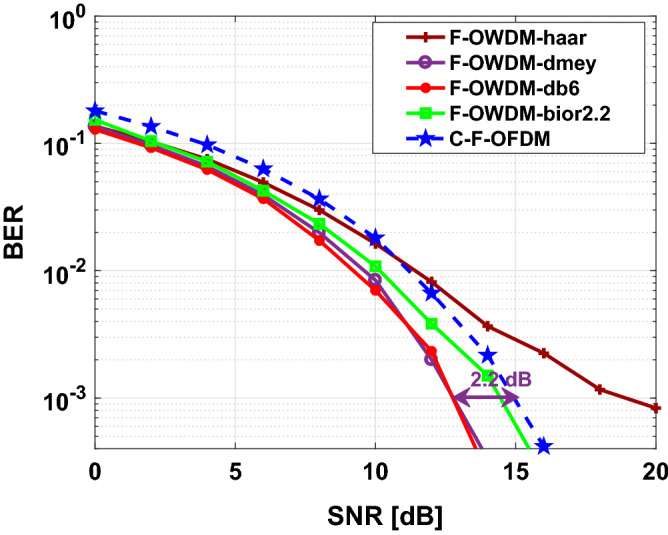
Comparison of BER of F-OWDM and C-F-OFDM signals based on the 16-QAM technique.

Figure 10 shows the comparison of the BER performance of the F-OWDM system with that of the C-F-OFDM system over an AWGN channel. In Fig. 10, the proposed F-OWDM system shows better BER performance than the C-F-OFDM system and shows 3 dB improvement in BER at $10^{-3}$ compared with C-F-OFDM. In this figure, all the wavelet show almost similar characteristics. However, the *haar* and *bior*2.2 wavelet surpasses other wavelets. Figure 11 shows, the BER vs. SNR plot for the 16-QAM modulation scheme. In this figure, the *dmey* and *db*6 wavelets show better performance compared to the other wavelets and indicate a gain of 2.2 dB in BER at $10^{-3}$.

It can be clearly seen in Figs. 10 and  11, that the BER characteristics are certainly worse for both the C-F-OFDM and F-OWDM systems as the modulation index changes from 4-QAM to 16-QAM. This indicates that the BER characteristics highly depend on the modulation index.

**Figure 12:**
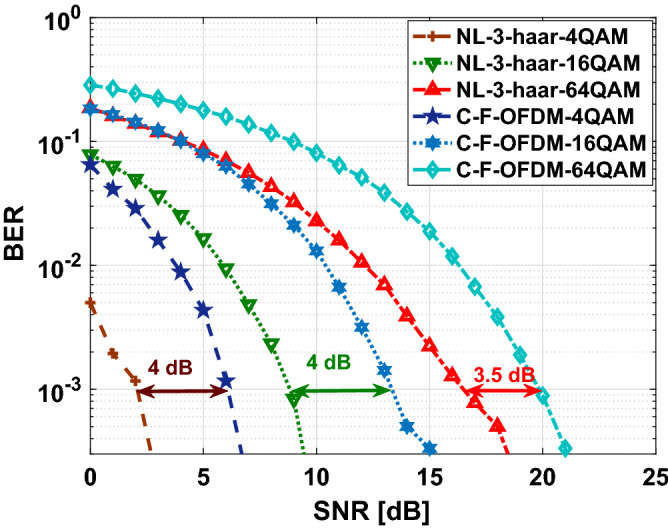
Comparison of BER of multilevel F-OWDM signal based on the $``haar''$ transform technique.

All the above mentioned analysis are based on a single decomposition/reconstruction level (NL=1). Theoretically, the maximum level of decomposition is referred to a level for which at least one coefficient is correct. Therefore, for an input signal of length *N*, the wavelet transform consists of $\log _{2} (N)$ decomposition levels as explained before. However, in practice, the maximum number of decomposition levels also depend on the choice of the mother wavelet. Hence, the maximum decomposition level can be calculated using the equation $NL=fix(\log _{2} (N)-\log _{2} (M-1))$, where M is the length of the mother wavelet, which depends on the type and order of mother wavelet^40^. Thus, in our analysis each wavelet model will have different maximum number of decomposition level. Hence, we chose single decomposition level (NL=1) for all our analysis. However, to study the effect of multilevel decomposition on the performance of F-OWDM system, we use 3-level *haar*-F-OWDM system and the effect of this multilevel decomposition on BER characteristics are demonstrated in Fig. 12.

Figure 12 shows, the comparison of the BERs of the proposed F-OWDM system for $NL=3$ decomposition. As illustrated in the figure, multilevel decomposition provides an improvement in BER performance in comparison with the C-F-OFDM system. Moreover, it is clear from the figure that, multilevel decomposition provides better BER characteristics compared to the single level F-OWDM system.

#### Rayleigh fading channel

The comparison of BER performance of F-OWDM and C-F-OFDM in flat Rayleigh fading channel with 10 channel taps is provided in this section. The received signal at the F-OWDM receiver can be represented as $r=Hy+\eta$, where $\eta$ represents additive white Gaussian noise and *H* is the transfer function of the channel impulse response. The simulation is accomplished using ZF channel equalization technique to retrieve the transmitted information. ZF equalizer refers to a form of linear equalization algorithm used in communication systems which applies the inverse of the frequency response of the channel to bringing down the ISI to zero in a noise free case.

**Figure 13:**
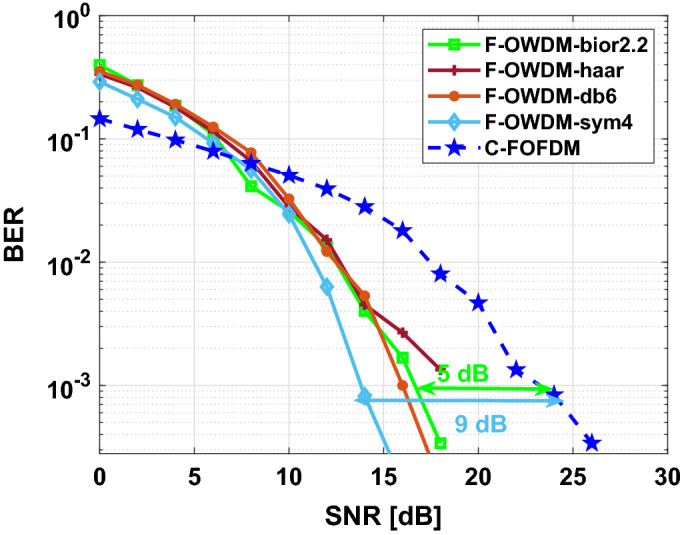
Comparison of BER of F-OWDM system under multipath channel for 4-QAM modulation.

**Figure 14:**
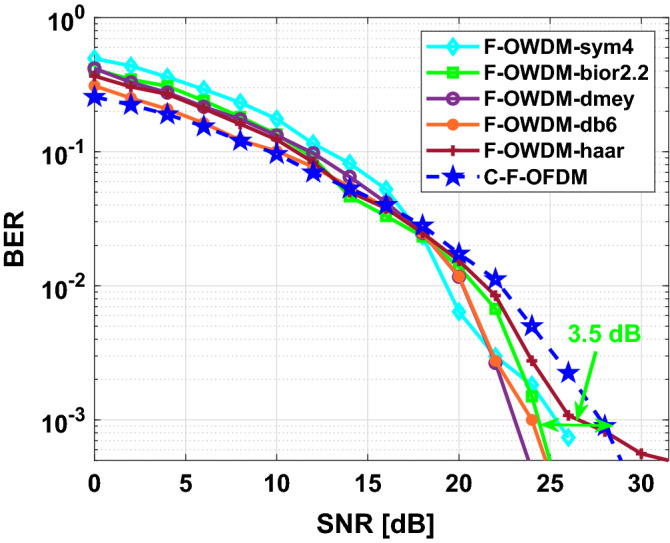
Comparison of BER of F-OWDM system under multipath channel for 16-QAM modulation.

Figure 13 shows the comparison of BER of the proposed F-OWDM system and C-F-OFDM over Rayleigh fading channel for the 4-QAM modulation scheme. As depicted in Fig. 13, *sym*4-F-OWDM and the *bior*2.2-F-OWDM provide better BER performance. The *sym*4-F-OWDM system attains 9 dB gain in terms of SNR for a BER of $10^{-3}$ and *bior*2.2-F-OWDM provides 5 dB gain, compared with C-F-OFDM. It is evident from the figure that, for low SNR values, the performance of all the wavelets are comparable to each other. Figure 14 shows the comparison of BER of the proposed F-OWDM system and C-F-OFDM over Rayleigh fading channel for the 16-QAM modulation scheme. As illustrated in Fig. 14, the *bior*2.2-F-OWDM attains a gain of 3.5 dB in terms of SNR for a BER of $10^{-3}$ compared with C-F-OFDM. For higher constellations (16-QAM) and for low SNR values, the performance of C-F-OFDM is comparable to that of the F-OWDM. However, for high SNR values, the proposed F-OWDM system surpasses C-F-OFDM system.

Thus, the following conclusions can be made from the analysis,

- Power spectral analysis: It is evident from the analysis that the *haar*-wavelet-based F-OWDM provides better PAPR characteristics compared to the other wavelets. However, the PSD of *dmey*-F-OWDM provides better spectral efficiency compared to those of the F-OWDM and C-F-OFDM systems based on all other wavelets. Thus, the choice of a suitable wavelet is limited by the trade-off between PAPR performance and PSD properties.
- PAPR performance: From the PAPR analysis, it is clear that all the wavelet modulation techniques used in F-OWDM attain better PAPR performance than C-F-OFDM. This means that the Fourier transform dissipates more energy compared to WT. However, out of all the different wavelets, *bior*2.2 and *Haar* wavelet provide lowest PAPR compared to C-F-OFDM. The PAPR characteristics of the F-OWDM system surpass the PAPR of the C-F-OFDM and C-OFDM system since, the F-OWDM system contains a smaller number of signal analysis than the C-F-OFDM system. The F-OWDM system considered only one-low pass filter region, and hence, there is lower probability for the peak to be above the average signals, leading to better PAPR characteristics. However, information at higher frequency branches may be lost due to the dyadic nature of decomposition. We can also conclude that the PAPR performance of wavelet modulation depends on the level of decomposition considered, the wavelet family selected and the modulation index.
- BER characteristics: From the simulation results, it is evident that all the chosen wavelets have almost the same BER performance under AWGN channel and deliver a gain of 3 dB at a BER of $10^{-3}$ compared to the C-F-OFDM. It can be also observed from the analysis that the error probability of both the proposed F-OWDM system and the C-F-OFDM systems increases as the number of constellation points increased. This means that the modulation index has an effect on BER performance as 4-QAM modulation provides improved BER characteristics compared with 16-QAM. It is also observed from our analysis that every wavelet based F-OWDM system has better performance compared to C-F-OFDM system over Rayleigh fading channel.
- According to our analysis, the *bior*2.2 and *haar* based F-OWDM shows better PAPR performance and BER characteristics. From the spectral analysis, it is clear that, all the wavelets have better PSD characteristics compared to C-F-OFDM. As explained before, the *haar* wavelet, which is the simplest wavelet function, is a memory efficient wavelet and preserves the energy of the signal. However, for the implementation of proposed F-OWDM system, we have considered *bior*2.2 instead of *haar* wavelet since, *haar* wavelet is highly discontinuous in nature causes high bandwidth usage for digital communication applications^41^.
  Furthermore, the other wavelet such as *db*6, *sym*4 also shows better performance. However, the data size of those wavelet families increases after IDWT operation, which requires more channel bandwidth. Meanwhile, when the complexity of the data and its rate increases Bi-orthogonal wavelet gives better performance. Moreover, Biorthogonal wavelets are more flexible and easy to design. Thus, Bi-orthogonal wavelet is the best choice for the implementation of our proposed F-OWDM system.
- Since the F-OWDM system does not use a CP, the data rate in F-OWDM will be higher than that in C-F-OFDM.

## Conclusion

The Filtered orthogonal wavelet division multiplexing system based on different wavelet techniques has been analyzed, and the performance of the proposed system is compared with that of C-F-OFDM system. In the F-OWDM system, the WT reconstitutes the Fourier transform block. Moreover, the proposed F-OWDM system does not use CP to avert the ICI and ISI. Thus, the F-OWDM system is able to save up to 25% of the bandwidth.

In this paper, the PAPR performance and the BER characteristics of the F-OWDM system are studied under multipath and AWGN channel. From the analysis, we were able to conclude that the F-OWDM system outperforms the C-F-OFDM system in term of BER and PAPR performance. In our study, a distinct number of wavelets are considered, and it is found that all wavelets achieve better PAPR performance compared to C-F-OFDM. Furthermore, the *bior*2.2 wavelet provides better PAPR characteristics and achieves almost similar spectrum characteristics as that of C-F-OFDM. Moreover, the BER characteristics of *bior*2.2-F-OWDM shows remarkable improvement compared to C-F-OFDM system over multipath fading channel. Thus, the *bior*2.2 wavelet-based-system is suggested as the best model for the proposed F-OWDM system due to its special bi-orthogonal characteristics.

From our analysis we can conclude that, F-OWDM might be a better alternative to C-F-OFDM for 5G and beyond applications since it has better bandwidth efficiency and better performance. Further investigation will be carried out to analyze the effect of wavelet-packet-modulation based MCM technique instead of simple WT.
